# Role of RING-Type E3 Ubiquitin Ligases in Inflammatory Signalling and Inflammatory Bowel Disease

**DOI:** 10.1155/2020/5310180

**Published:** 2020-08-10

**Authors:** Liguo Zhu, Ying Li, Longyuan Zhou, Guang Yang, Ying Wang, Jing Han, Li Li, Shenghong Zhang

**Affiliations:** Division of Gastroenterology, The First Affiliated Hospital, Sun Yat-sen University, Guangzhou, China

## Abstract

Ubiquitination is a three-step enzymatic cascade for posttranslational protein modification. It includes the ubiquitin-activating enzyme (E1), ubiquitin-conjugating enzyme (E2), and ubiquitin ligase (E3). RING-type E3 ubiquitin ligases catalyse the posttranslational proteolytic and nonproteolytic functions in various physiological and pathological processes, such as inflammation-associated signal transduction. Resulting from the diversity of substrates and functional mechanisms, RING-type ligases regulate microbe recognition and inflammation by being involved in multiple inflammatory signalling pathways. These processes also occur in autoimmune diseases, especially inflammatory bowel disease (IBD). To understand the importance of RING-type ligases in inflammation, we have discussed their functional mechanisms in multiple inflammation-associated pathways and correlation between RING-type ligases and IBD. Owing to the limited data on the biology of RING-type ligases, there is an urgent need to analyse their potential as biomarkers and therapeutic targets in IBD in the future.

## 1. Introduction

Ubiquitination is a crucial part of a diverse range of physiological and pathological processes, such as protein degradation and inflammation-associated signalling [1, 2]. It is a three-step enzymatic process that consists of ubiquitin-activating enzyme (E1), ubiquitin-conjugating enzyme (E2), and ubiquitin ligase (E3) [3]. E3 ligases transfer activated ubiquitin from E2 to specific substrates, thereby forming mono- or polyubiquitinated proteins to activate proteasome-mediated proteolysis, signal transduction, endocytosis, etc. [3]. E3 ligases are crucial as they catalyse target ubiquitination and enable the formation of polyubiquitin chains that enhance the complexity of ubiquitination in physiological and pathological processes. Although dysregulated ubiquitination is involved in the development of various types of immune pathologies (e.g., systemic lupus erythematosus, rheumatoid arthritis, and inflammatory bowel disease [IBD]) [1, 4], there is limited knowledge regarding the role of RING-type ligases in inflammation-associated pathways. In this review, we have focused on IBD owing to its complex pathogenesis involvement in a wide range of etiological factors, including dysregulated ubiquitination. The term IBD is used for a group of chronic autoimmune gastrointestinal disorders, including mainly Crohn's disease (CD) and ulcerative colitis (UC) [5]. Chronicity of IBD often causes intestinal complications, hospitalisation, steroid dependency, and surgery in diagnosed patients [6]. Although significant progress has been made in understanding the nature of IBD, the underlying interacting mechanism involving ubiquitination, RING-type ligases, and onset of IBD remains to be fully understood.

## 2. Ubiquitination Mediated by RING-Type Ligases

**Figure 1:**
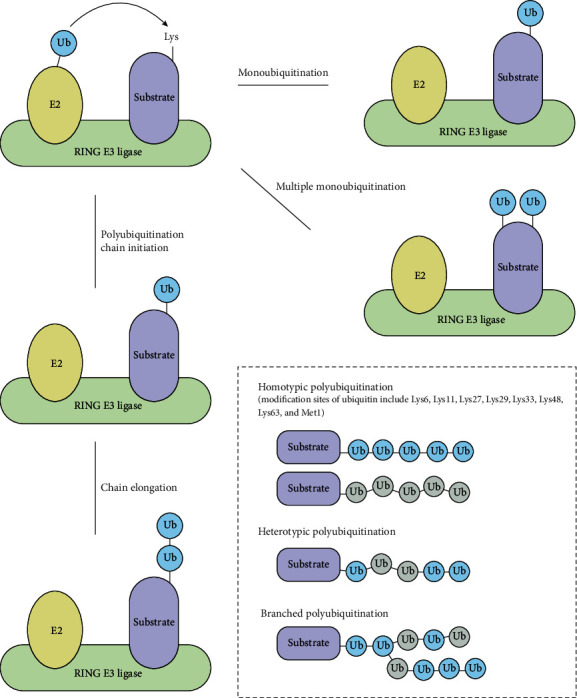
Functional mechanisms of RING-type E3 ligase. Acting as a scaffolding, RING-type E3 ligase recruits a E2-ubiquitin thioester and a substrate and allows the lysine of substrate to attack the thioester for ubiquitin transfer. RING-type E3 ligases catalyse monoubiquitination or multiple monoubiquitination by transferring a single ubiquitin to one or several residues of the substrate. For polyubiquitination, ubiquitins form eight different linkage types including Met1, Lys6, Lys11, Lys27, Lys29, Lys33, Lys48, and Lys63. Apart from homotypic chains, RING-type E3 ligases also catalyse heterotypic chains and branched ubiquitin chains by adopting multiple linkage types and branched topology in the formation of polyubiquitin chains.

Human cells express more than 600 E3 ubiquitin ligases that are classified into three types based on their catalytic domains: RING, HECT (homologous to the E6AP carboxyl terminus), and a recently identified RBR- (RING-between RING-RING-) type of E3 ligases [7, 8]. The RING domain has a crossbraced structure with two atoms of zinc that catalyse the direct transfer of ubiquitin from the E2-Ubiquitin thioester to the substrate [9] (Figure 1). Apart from catalysing monoubiquitination, RING-type E3 ligases also elongate homotypic polyubiquitin chains with varying linkage specificities, such as that on Lys48 during the proteasomal targeting of substrates and Lys63 in signal transduction, thereby modulating proteins for proteolytic and nonproteolytic activity [10]. However, their roles in catalysing other (less common) types of ubiquitination, including atypical homotypic (e.g., Lys6, Lys11, Lys27, Lys29, Lys33, and Met1 [11]), heterotypic, and branched polyubiquitination, remain ambiguous. The efficiency of RING-type E3 ligases in ubiquitination depends on multiple factors, such as substrate modification, phosphorylation of E2/E3 enzymes, autoubiquitination by E3 ligases, and pseudosubstrate competition [10]. The role of the RING-type ligases and their sophisticated functional mechanism of ubiquitination will be discussed in the following sections.

## 3. Signalling Pathways Regulated by RING-Type Ligases

### 3.1. Pathogen Recognition

Under physiological conditions, pattern recognition receptors (PRRs) comprise toll-like receptors (TLRs), retinoic acid inducible gene- (RIG-) I-like receptors (RLRs), C-type lectin-like receptors, and nucleotide-binding oligomerisation domain-like receptors. PRRs recognise pathogen-associated molecular patterns (PAMPs) and trigger the activation of downstream effectors in innate immune responses [12]. For inflammatory diseases that are closely associated with microbiome dysbiosis, such as IBD [13, 14], dysregulation of PRRs and relevant RING-type ligases may be involved in pathogen-induced inflammation.

#### 3.1.1. TLR Signalling

Upon recognising a wide range of microbial components, such as lipopolysaccharides, flagellin, and microbial nucleic acids, activated TLRs expressed on antigen-presenting cells trigger effector T cell responses in inflammatory diseases [15–17]. RING-type ligases modulate the activation of PAMP-induced TLRs. By directly binding with TLR3, ring finger protein 170 (RNF170) catalyses the Lys48-linked ubiquitination and proteasomal degradation of TLR3, thereby suppressing TLR3-mediated innate immunity in macrophages [18]. On the other hand, Fc*γ* receptor (Fc*γ*R) IIb, an inhibitory FcR on antibody-dependent monocyte phagocytosis, is targeted by MARCH3 (RNF173) for ubiquitination and degradation in lipopolysaccharide-induced TLR4 activation [19].

#### 3.1.2. RIG-I Signalling

RIG-I is a cytoplasmic PRR that recognises viral RNA and triggers the activation of downstream immune responses that are associated with both viral infections and noninfectious autoimmune diseases, such as enterocolitis [20]. The deficiency of RIG-I aggravates virus-induced cell death in intestinal epithelial cells and induces susceptibility to chemically induced colitis in mice, suggesting the importance of RIG-I signalling in intestinal antiviral immune response [20, 21]. E3 ligases, like RNF122 and RNF125, mediate Lys48-linked RIG-I ubiquitination and proteasomal degradation, leading to the reduced expression of infection induced-proinflammatory cytokines, including IL-6 and type I interferons (*α* and *β*) [22, 23]. In contrast, independent of its E3 ligase activity, RNF123 binds with the CARD domain of RIG-I and melanoma differentiation-associated gene 5 to compete with the mutual downstream adaptor mitochondrial antiviral signalling protein (MAVS) and inhibit RLR-mediated antiviral response [24]. Unlike the aforementioned RING-type ligases that directly target RLRs, RNF114 negatively regulates RLR signalling by polyubiquitinating and inducing the proteasomal degradation of MAVS [25].

### 3.2. Proinflammatory Pathways

#### 3.2.1. Nuclear Factor Kappa B (NF-*κ*B) Signalling

The NF-*κ*B pathway is one of the most well-studied proinflammatory pathways regulated by ubiquitination [26]. TRAF6 (RNF85) ubiquitinates the evolutionarily conserved signalling intermediate in TLR activation that is essential for TLR4-dependent NF-*κ*B activation [27]. RNF183 promotes NF-*κ*B signalling by inducing the ubiquitin-dependent degradation of I*κ*B*α* [28]. TRAF2 (RNF117) and TRAF3 (RNF118) induce Lys48-linked ubiquitination and proteasomal degradation of c-Rel and interferon regulatory factor 5, thereby prohibiting the synthesis of proinflammatory cytokines in macrophages [29]. MKRN2 (RNF62) mediates the polyubiquitination and degradation of the p65 subunit of NF-*κ*B, thereby inhibiting NF-*κ*B signalling [30]. RNF114 negatively regulates NF-*κ*B signalling and T cell activation by ubiquitinating and stabilising NF-*κ*B signalling inhibitors A20 and I*κ*B*α* [31, 32]. It has also been reported that RNF20 downregulation decreases histone H2B monoubiquitination and leads to the NF-*κ*B-dependent transcription of proinflammatory cytokines, such as IL-6 and IL-8 [33]. Nevertheless, despite the formation of heterodimers of RNF40 with RNF20, RNF40 alone activates NF-*κ*B signalling and upregulates NF-*κ*B-dependent transcription by promoting I*κ*B kinase (IKK) phosphorylation and p65 nuclear translocation, indicating the involvement of NF-*κ*B-dependent transcription in the ubiquitination of substrates other than H2B [34]. MARCH3 (RNF173) mediates Lys48-linked ubiquitination and lysosomal degradation of IL-1 receptor I and thereby inhibits IL-1*β*-triggered NF-*κ*B activation [35]. Independent of its E3 ligase activity, RNF8 inhibits TNF-*α*-induced NF-*κ*B activation by directly binding with the kinase domain of IKK and interfering with IKK*α*/*β* phosphorylation [36]. RNF11 also exerts a noncanonical role in negatively regulating NF-*κ*B signalling. RNF11 has high affinity for the E2 enzyme Ubc13 and minimal E3 ligase activity that subsequently outcompetes E1 enzymes and other E3 enzymes, such as TRAF6 [37], and impedes the activation of NF-*κ*B signalling [38].

#### 3.2.2. Mitogen-Activated Protein Kinase (MAPK) Signalling

MAPKs are another family of proteins closely related to inflammation-associated pathologies, such as IBD [39]. Upon stimulation by TNF-*α*, TRAF2 is autoubiquitinated on the Lys63 residue that enables its translocation to the cytoskeletal fraction and activates JNK signalling [40, 41]. *In vitro* experiments have shown that JNK signalling is suppressed and enhanced in cells overexpressing RNF213 and RNF186, respectively [42, 43]; however, the mechanisms involved in this regulation remain to be understood. TRAF7 (RNF119) upregulates the kinase activity of mitogen-activated protein kinase 3 via the WD40 domain and potentiates cell apoptosis via the RING finger domain [44]. Similarly, RNF13 mediates endoplasmic reticulum (ER) stress-induced JNK activation and subsequent cell apoptosis by binding with and promoting the phosphorylation of the ER stress sensor endoplasmic reticulum to nucleus signalling 1 [45].

#### 3.2.3. Janus Kinase (JAK)/Signal Transducer and Activator of Transcription 3 (STAT3) Signalling

JAK/STAT3 is one of the major proinflammatory signalling pathways that orchestrate the progression of inflammatory and autoimmune diseases [46]. A number of RING-type ligases modulate JAK/STAT3 signalling. RNF6 and RNF38 function in catalysing the ubiquitination-induced proteasomal degradation of SH2-containing protein tyrosine phosphatase 1 that targets phosphorylated STAT3, thereby maintaining STAT3 phosphorylation and activating STAT3 signalling [47, 48]. In contrast, TRAF6 promotes Lys63-linked ubiquitination of STAT3 and represses STAT3-mediated transcription of downstream inflammation-related genes, such as C-reactive protein [49]. Interestingly, RNF41 modulates the cell surface expression of JAK2-associated cytokine receptors by blocking the cleavage of receptors and enhancing receptor shedding in a ubiquitination-dependent manner [50].

#### 3.2.4. Phosphatidylinositol 3-Kinase (PI3K) Signalling

PI3K is another classical pathway involved in inflammation wherein RING-type ligases are of crucial importance. MKRN1 (RNF61) functions in the positive-feedback regulation of sustained PI3K/AKT activation upon stimulation by epidermal growth factor: AKT activation phosphorylates and stabilises E3 ligase MKRN1 that further ubiquitinates and degrades phosphatase and tensin homologue (a PI3K/AKT inhibitor) [51]. MKRN2 (RNF62) induces the ubiquitin-dependent degradation of the p85*α* subunit of PI3K and downregulated AKT phosphorylation, suggesting a negative regulatory role of MKRN2 in PI3K/AKT signalling [52]. Downregulation of UHRF1 (RNF106) represses the phosphorylation of PI3K and AKT, which reveals an underlying interaction between UHRF1 and PI3K/AKT signalling [53].

### 3.3. Transforming Growth Factor-*β* (TGF-*β*) Signalling

TGF-*β* signalling functions in immunosuppression and inhibiting the activity of effector T cells, maintaining T_reg_ differentiation, reducing B cell responsiveness, and inducing macrophage anergy [54]. RNF11 plays a dual role in the modulation of TGF-*β* signalling. By competing with Smad7 for Smurf2, RNF11 is a positive regulator for TGF-*β* signalling and reduces the formation of Smad7/Smurf2 complexes that degrade TGF-*β* receptors [55]. RNF11 is also responsible for the ubiquitination-mediated stabilisation of Smad4 that enhances Smad4-dependent TGF-*β* signalling by direct interaction [56]. Notably, RNF11 may negatively regulate TGF-*β* signalling by enabling the formation of Smurf2/RNF11 complexes and inducing the ubiquitination and degradation of the associated molecule with the SH3 domain of STAM that promotes TGF-*β* signalling [57]. PRAJA (RNF70) mediates the ubiquitination-induced proteasomal degradation of embryonic liver fodrin (a Smad4 adaptor protein), thereby negatively regulating TGF-*β* signalling [58].

### 3.4. Autophagy

Accumulating evidence reveals that autophagy contributes extensively to immune cell development and cell death, and its dysregulation has been implicated in many autoimmune diseases [59]. TRAF6 catalyses Lys63-linked ubiquitination of BECN1 and stimulates TLR-induced autophagy in macrophages upon proinflammatory stimulation [27, 60]. RNF166 has a novel role in antibacterial host defence owing to its function in inducing the Lys29- and Lys33-linked ubiquitination of autophagy adaptor p62, which mediates the recruitment of p62 to bacteria and initiates bacteria engulfment [61].

### 3.5. Noncoding RNAs

Since dysregulated noncoding RNAs are involved in the progression of inflammatory diseases [62], the posttranscriptional regulation of RING-type ligases by noncoding RNAs may play critical roles in potential inflammation-relevant signalling pathways. Until now, quite a few microRNAs have been proved to posttranscriptionally regulate RING-type ligases by hampering translation or inducing mRNA degradation (Table 1) [28, 63–71]. Nevertheless, although the other two major types of noncoding RNAs (long noncoding RNAs and circular RNAs) also have diverse functions in inflammatory diseases (e.g., competing endogenous RNA [ceRNA], transcription regulation, and RNA-binding protein sponges) [72, 73], to what extent they modulate RING-type ligases awaits further analysis.

## 4. RING-Type Ligases in IBD

### 4.1. Pathogenesis of IBD

The pathogenesis of IBD has been elucidated over the past years. More than 200 loci have been implicated in increased genetic risk for IBD that correlate with the functioning of cellular processes, such as innate/adaptive immune response, intestinal mucosal barrier homeostasis, and autophagy, suggesting the involvement of multiple factors in shaping the procolitogenic environment during the development of IBD [74, 75]. IBD patients manifest with abnormalities in the composition of gut microbiota, such as decreased bacterial diversity, increased proportion of harmful bacterial strains, and decreased proportion of protective probiotics, which trigger proinflammatory intestinal pathogenic immune responses and contribute to the pathogenesis of IBD [14, 76]. Elevated levels of proinflammatory cytokines (e.g., IL-1, IL-6, and IL-23) and activation of adaptive (e.g., Th1, Th2, Th9, and Th17 cells) and innate immune cells (e.g., neutrophils and NK cells) constitute a synergistic inflammatory network that induces intestinal mucosal inflammation and sustained activation of multiple proinflammatory signalling pathways [17, 77]. IL-1 family-induced NF-*κ*B, IL-6-induced STAT3, MAPK, and PI3K signalling pathways are pivotal in intestinal inflammation [39, 78, 79]. TGF-*β* signalling can mitigate immune cell hyperactivation but also causes the formation of intestinal strictures in chronic intestinal inflammation [54]. Autophagy is involved in the regulation of immune cell function; thus, defective autophagy also plays an important role in IBD pathogenesis [59]. A dysfunctional gut barrier and subsequent increased intestinal permeability are also considered important etiologic factors in the development of IBD that result in the uncontrolled exchange of materials between the intestinal lumen and internal environment. A compromised gut barrier is often attributed to the proinflammatory stimulation and subsequent downregulation of sealing tight junction proteins (e.g., claudin-5 and claudin-8) and upregulation of pore-forming tight junction proteins (e.g., claudin-2) [80–83]. Since multiple etiologic factors function in the pathogenesis of IBD in a synergistic manner, further research is warranted to understand the dynamics between these players.

### 4.2. Role of RING-Type Ligases in IBD

Despite the limited knowledge on RING-type ligases in IBD, research suggests a correlation between RING-type ligases and IBD pathogenesis (Table 2). Genome-wide association studies have identified RNF186 as one of the genes associated with susceptibility to UC; the disease-coding variant of RNF186 involves an altered RING domain [84, 85]. The truncated RNF186 lacking the second transmembrane domain is associated with protecting individuals against developing UC by inhibiting the ER localisation of RNF186 and subsequent Lys29- and Lys63-linked polyubiquitination of proapoptotic BCL2 interacting protein 1 under ER stress [86, 87]. Notably, RNF186 functions differently in a dextran sulfate sodium- (DSS-) induced mouse model of colitis: RNF186-deficient mice develop more severe colitis during DSS administration, and their colonic epithelial cell exhibits enhanced signs of ER stress and apoptosis [88]. RNF186 also modulates intestinal barrier function by mediating the Lys48-linked ubiquitination of tight junction protein occludin, and RNF186 deficiency increases intestinal permeability in RNF186 knockout mice [88]. Thus, RNF186 targets different substrates and has a complex association with gut inflammation.

Apart from the reduced expression of RNF20 in the colon samples from UC patients, homozygous RNF20-knockout mice die due to embryonic lethality, and heterozygous mice are susceptible to DSS-induced colitis with increased intestinal permeability, suggesting the anti-inflammatory role of RNF20 [33]. RNF40 knockout mice exhibit mitigated gut inflammation upon treatment with DSS; this can be attributed to the attenuated activation of NF-*κ*B signalling [34]. The upregulation of RNF183 in the colonic tissue from IBD patients and 2,4,6-trinitrobenzenesulfonic acid- (TNBS-) induced mouse model of colitis indicates its proinflammatory function, probably by promoting the ubiquitination-induced degradation of I*κ*B*α* [28].

Because UHRF1-catalysed histone H3 monoubiquitination recruits and stimulates DNA methyltransferase 1 to DNA methylation sites, and thereby maintains DNA methylation, UHRF1 participates in the epigenetic control of multiple genes, such as TNF-*α* [89, 90]. Mice with macrophages deficient for UHRF1 manifest with TNF-*α* overexpression and aggravated DSS-induced colitis. Also, the loss of function in UHRF1 reduces the methylation of the TNF-*α* promoter in macrophages, indicating the regulatory role of UHRF1 in the mouse model of colitis [91]. Similarly, an *in vivo* study in zebrafish has revealed that loss in function of UHRF1 leads to defects in the epigenetic regulation of TNF-*α* promoter methylation and elicits elevated TNF-*α* expression in inflammatory processes, including intestinal epithelial cell apoptosis, neutrophil recruitment, and weakened intestinal barrier function [92]. However, UHRF1 may effect differently among subtypes of regulatory T (T_reg_) cells, as UHRF1 maintains the proliferation and maturation of colonic T_reg_ cells but inhibits the differentiation of peripheral induced T_reg_ cells in the development of colitis [93, 94].

Studies have shown the diverse roles involved with the upregulation of TRAFs, including TRAF1/2/3/5, in the blood or colonic mucosa of IBD patients [95, 96]. DSS-induced colitis models of TRAF2- and TRAF3-deficient mice reveal similar functions of TRAF2 and TRAF3 as negative regulators of experimental colitis by decreasing proinflammatory cytokines and reducing the infiltration of immune cells in the colon [29]. In another study, TRAF2-deficient mice develop severe spontaneous colitis and exhibit altered colonic microbiota composition, indicating the anti-inflammatory role of TRAF2 in controlling colonic microbiota [97]. TRAF3 also acts as a colitis regulator by binding with the IL-17 receptor and interfering with the IL-17-mediated proinflammatory pathway in mice with TNBS-induced colitis [98]. Although TRAF5 (RNF84) promotes the ubiquitination and stabilisation of the retinoic acid-related orphan receptor *γ*t that mediates proinflammatory Th17 cell differentiation and IL17A/IL17F expression [99, 100], TRAF5-deficient mice exhibit aggravated experimental colitis and upregulation of proinflammatory cytokines [101]. The complex function of TRAF5 needs further analysis.

## 5. Discussion

As the importance of RING-type E3 ligases is gradually unveiled, there are still problems to be solved. Firstly, apart from the canonical role of RING-type ligases in modulating key signalling pathways and their downstream adaptors as E3 ubiquitin ligases, some RING-type ligases interfere with the ubiquitination cascade by competition or direct interaction with other E3 ligases [102]. Owing to the variety of RING-type ligases and substrate specificity of E3 ligases, the potential competition among RING-type ligases in regulating immune response remains to be fully understood. Secondly, differences in RING E3 ligase-mediated target ubiquitination can also be attributed to the variance in length and linkage type of ubiquitin chains. Although RING-type ligases function in proteolytic degradation and signal transduction by catalysing Lys48-linked and Lys63-linked ubiquitination, respectively, their roles in catalysing less common linkage types of homotypic polyubiquitin chains, such as Lys11-linked ubiquitination [103], and the outcome of such polyubiquitination are still obscure.

RING-type ligases form a sophisticated but important ubiquitination network, wherein the expression and function of RING-type ligases are also influenced reciprocally in physiological and pathological processes. Understanding the mechanisms employed by RING-type ligases in modulating inflammation-associated pathways by catalysing atypical linkages and affecting signal transduction may further explain the interaction between RING-type ligases and IBD. Similarly, research on heterotypic polyubiquitin chains is also important to unveil these underlying mechanisms.

In this review, we have highlighted the roles of RING-type ligases in PAMP recognition and modulation of inflammation-associated pathways that are crucial etiological factors in the development of autoimmune diseases. Accumulating evidence shows that many RING-type ligases are involved in inflammation-associated pathways, such as proinflammatory NF-*κ*B, MAPK, JAK/STAT3, and PI3K signalling and anti-inflammatory TGF-*β* signalling. Subsequently, we have discussed the role of RING-type ligases in the pathogenesis of IBD via inflammation-related pathways. Patients with IBD exhibit the differential expression of specific RING-type ligases, such as TRAFs. However, there are limited studies on the potential clinical value of RING-type ligases in predicting or treating IBD. Thus far, there have been a few attempts to use RING-type ligases as predictive biomarkers and therapeutic targets in treating cancer; RNF43 modulates Wnt signalling and has been used to target colorectal cancer and pancreatic ductal carcinoma [104, 105]. Nevertheless, the potential of RING-type ligases in autoimmune diseases, especially in IBD, needs to be understood in greater detail. Therefore, future research on the expression profile of RING-type ligases in the gastrointestinal tract and the detailed mechanisms is warranted.

## Figures and Tables

**Table 1:** RING-type ligases targeted by noncoding RNAs and the relevant pathways.

RING-type ligase	Noncoding RNA	Relevant pathway	Reference
RNF11	miR-19b-3p	NF-*κ*B signalling	[63]
RNF183	miR-7	NF-*κ*B signalling	[28]
		ER stress-induced apoptosis	[64]
RNF2	miR-139-5p	MAPK signalling	[65]
RNF135	miR-485-3p	MAPK/ERK signalling	[66]
RNF125	miR-15b	RIG-I signalling	[67]
TRAF6	miR-124	TLR signalling	[68]
	miR-146	TLR signalling	[69]
c-Cbl	miR-216a	PI3K/AKT signalling	[70]
ZNRF2	lncRNA TTN antisense RNA 1 acts as a ceRNA for miR-153-3p	PI3K/AKT signalling	[71]

**Table 2:** Roles of RING-type ligases in IBD patients and animal models of colitis.

RING-type ligase	Role	IBD patients	Animal model of colitis	Reference
RNF186	Controversial	Increase risk of UC and ER stress-induced apoptosis	Attenuate ER stress and maintain intestinal permeability in DSS-induced mouse model of colitis	[84–88]
RNF20	Anti-inflammatory	Decrease in the colonic tissue from UC patients	Protect mice from DSS-induced colitis and maintain intestinal barrier	[33]
RNF40	Proinflammatory	—	Activate NF-*κ*B signalling in DSS-induced mouse model of colitis	[34]
RNF183	Proinflammatory	Increase in the colonic tissue from CD and UC patients correlate with endoscopic index of disease severity	Increase in TNBS-induced mouse model of colitis	[28]
UHRF1	Anti-inflammatory	—	Regulate TNF-*α* expression in mice with DSS-induced colitis and zebrafish Modulate the proliferation and differentiation of T_reg_ cells	[89–94]
TRAF2	Anti-inflammatory	Increase in the colonic tissue from CD and UC patients	Modulate colonic microbiota composition, proinflammatory cytokine expression, and immune cell infiltration in mice with DSS-induced colitis	[29, 96, 97]
TRAF3	Anti-inflammatory	Increase in the plasma and colonic tissue from CD and UC patients	Regulate proinflammatory cytokine expression and mitigate inflammatory damage in DSS-induced mouse model of colitis Inhibit IL-17-mediated proinflammatory pathway in TNBS-induced mouse model of colitis	[29, 95, 98]
TRAF5	Anti-inflammatory	Increase in the plasma and colonic tissue from CD and UC patients	Control proinflammatory cytokine expression and protect mice against experimental colitis	[99–101]
